# Metabolic Imaging of Hyperpolarized [1-^13^C]Pyruvate in a Ferret Model of Traumatic Brain Injury

**DOI:** 10.3390/ijms26115327

**Published:** 2025-06-01

**Authors:** Dirk Mayer, Abubakr Eldirdiri, Amanda L. Hrdlick, Boris Piskoun, Joshua C. Rogers, Aditya Jhajharia, Minjie Zhu, Julie L. Proctor, Ulrich H. Leiste, William L. Fourney, Jody C. Cantu, Gary Fiskum, Molly J. Goodfellow

**Affiliations:** 1 Department of Diagnostic Radiology and Nuclear Medicine, University of Maryland, Baltimore, MD 21201, USA; aeldirdiri@som.umaryland.edu (A.E.); jcrogers@som.umaryland.edu (J.C.R.); ajhajharia@som.umaryland.edu (A.J.); mjzhu@terpmail.umd.edu (M.Z.); 2 Fischell Department of Bioengineering, University of Maryland, College Park, MD 20742, USA; 3 Department of Anesthesiology, University of Maryland, Baltimore, MD 21201, USA; ahrdlick@som.umaryland.edu (A.L.H.); bpiskoun@som.umaryland.edu (B.P.); jproctor@som.umaryland.edu (J.L.P.); gfiskum@som.umaryland.edu (G.F.); mgoodfellow@som.umaryland.edu (M.J.G.); 4 Department of Aerospace Engineering, University of Maryland School of Engineering, College Park, MD 20742, USA; uleiste@umd.edu (U.H.L.); four@umd.edu (W.L.F.); 5 Air Force Research Laboratory, 711th Human Performance Wing, Air and Space Biosciences Division, En Route Care Section, US Air Force Materiel Command, Baltimore, MD 20723, USA; jcantu@som.umaryland.edu

**Keywords:** traumatic brain injury, ferret, hyperpolarized ^13^C pyruvate, metabolic imaging, energy metabolism

## Abstract

It is increasingly recognized that early perturbation of energy metabolism might have important implications in management and ultimately the neurological outcome in patients with traumatic brain injury (TBI). At the same time, treatments and screening tools successfully developed in preclinical TBI models have failed to translate to the clinic. As ferrets possess primate-like gyrencephalic brains that may better replicate the human response to neurologic injury, the goal of this study was to noninvasively measure brain energy metabolism after injury in a ferret model of TBI. To this end, metabolic imaging of hyperpolarized (HP) [1-^13^C]pyruvate (Pyr) and its conversion to lactate (Lac) and bicarbonate (Bic) was performed in ferrets before and after combined under-vehicle blast and controlled cortical impact injury. Reduced Bic/Pyr, reflecting reduced pyruvate dehydrogenase activity, was detected 8–10 days post-injury whereas no difference in Lac/Pyr was observed. These results demonstrate the feasibility of using metabolic imaging of HP [1-^13^C]Pyr to measure perturbations in brain energy metabolism in a novel highly translatable animal model of TBI. The method may contribute to both improved understanding of injury mechanisms and more effective drug development.

## 1. Introduction

Traumatic brain injury (TBI) is a leading cause of death and disability, affecting an estimated 1.7 million people in the United States annually [1]. TBI survivors can face lasting disability, including cognitive impairments, and are at increased risk for mood disorders and neurological/neurodegenerative disease [2,3]. Unfortunately, clinically utilized imaging techniques tend to underestimate brain damage in mild-to-moderate TBI [4], making the objective detection and accurate characterization of these injuries an unmet clinical need. 

The initial brain injury can induce a number of secondary insults including deranged energy metabolism. The relative increase in anaerobic over oxidative metabolism may have implications for treatment and prognosis [5,6,7,8,9]. Hyperpolarized (HP) ^13^C magnetic resonance imaging (MRI) allows for real-time, noninvasive imaging of metabolic pathways in vivo [10]. Pyruvate (Pyr), which links glycolysis to the tricarboxylic acid (TCA) cycle, is an ideal substrate to probe brain energy metabolism and has already been applied in rodent TBI models [11,12,13,14]. However, the clinical translation of rodent studies is limited by the structural differences between the lissencephalic rodent and gyrencephalic human brains. For example, the mechanical stress from impact is evenly distributed on the lissencephalic brain whereas the presence of sulci focuses mechanical stress to the sulcal base [15]. This may produce differential effects including the location and severity of injury. This may, in turn, influence pathophysiology (e.g., differing cell types in brain nuclei have varied metabolic profiles [16]) and neurobehavioral outcomes. The white–gray matter ratio is also important as the white matter is particularly sensitive to injury in humans (e.g., diffuse axonal injury, edema) and white matter metabolism significantly differs from that of gray matter [16,17]. Ferrets possess primate-like brains including similar cerebrovascular anatomy, a gyrencephalic cortex, and a high white–gray matter ratio. These features may better recapitulate the human brain response to trauma and, thereby, enhance clinical translation [18,19]. Therefore, the aim of this study was to investigate the feasibility of metabolic imaging of HP [1-^13^C]Pyr in a ferret TBI model to noninvasively assess perturbations in brain energy metabolism after combined under-vehicle blast and controlled cortical impact (bCCI).

## 2. Results

**Figure 1:**
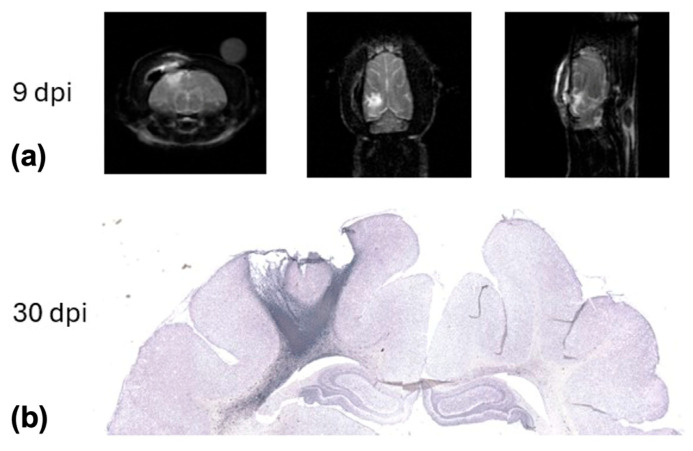
(**a**) T2-weighted MRI in all three principal orientations from a representative animal 9 days after bCCI injury illustrating hyperintense signal in the cerebral cortex due to edema below the impact site. (**b**) Photomicrograph (5× montage) of immunohistochemical detection of microglia (Iba1) in the same animal, euthanized 30 days post-injury. The image depicts missing/fenestrated tissue at the impact site as well as marked Iba1 immunoreactivity, particularly in the white matter. (MRI: magnetic resonance imaging; bCCI: blast and controlled cortical impact; dpi: days post injury; iba1: ionized calcium-binding adaptor molecule 1).

Injury was confirmed in T2-weighted ^1^H-MRI (Figure 1a) by the presence of hyperintensity near the location of impact as well as through histology (Figure 1b) by missing/fenestrated tissue at the impact site.

**Figure 2:**
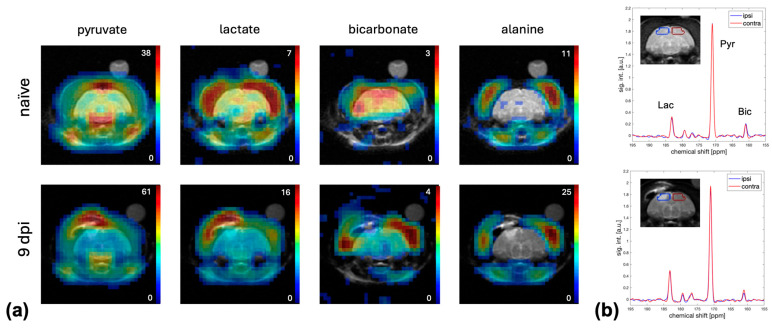
(**a**) Metabolic maps with a 60 × 60 mm^2^ FOV of Pyr, Lac, Bic, and Ala from an animal before (**top**) and 9 days after (**bottom**) bCCI injury superimposed on the corresponding T2-weighted MRI. The urea phantom used for calibration is visible in the MRI on top of the ferret head. The intensity scales next to each map indicate the absolute signal intensity for the respective metabolite in arbitrary units. (**b**) Corresponding ^13^C spectra from ROIs in the ipsilateral (blue) and contralateral (red) hemisphere of the animal before (**top**) and after (**bottom**) injury. The location of the ROIs is indicated on the ^1^H MRI inlets. Both the Bic map and the spectra from the animal after injury show lower Bic in the ipsilateral compared to contralateral hemisphere. (FOV: field-of-view; Pyr: pyruvate; Lac: lactate; Bic: bicarbonate; Ala: alanine; MRI: magnetic resonance imaging; bCCI: blast and controlled cortical impact; dpi: days post injury; ROI: region of interest).

Metabolic maps of Pyr, lactate (Lac), bicarbonate (Bic), and alanine (Ala) superimposed onto the corresponding T2-weighted MRI from a representative animal before and 9 days post-injury are shown in Figure 2. In the naïve animal, the highest Pyr signal was detected in vasculature structures such as the circle of Willis and the sagittal sinus. Pyr and its metabolic products were also detected in the thick muscle tissue surrounding the skull with Lac and Bic also detected in the brain. In contrast, little to no Ala was observed in the brain. Also shown in Figure 2 are the corresponding spectra averaged over the ipsilateral and contralateral brain regions of interest (ROIs).

**Figure 3:**
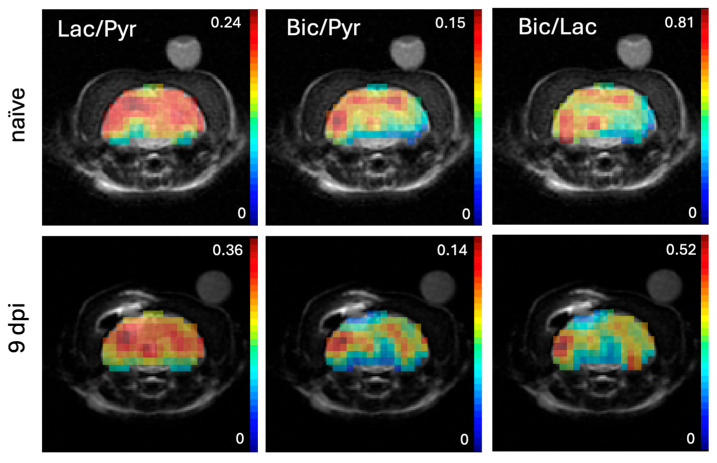
Metabolic maps masked to the region of the brain of Lac/Pyr, Bic/Pyr, and Bic/Lac from the same animal as shown in Figure 2 before (**top**) and 9 days after (**bottom**) bCCI injury superimposed on the corresponding T2-weighted MRI. Lower signal intensity at the location of T2 hyperintensity is seen in both the Bic/Pyr and Bic/Lac maps after injury. (Pyr: pyruvate; Lac: lactate; Bic: bicarbonate; MRI: magnetic resonance imaging; bCCI: blast and controlled cortical impact; dpi: days post injury).

**Figure 4:**
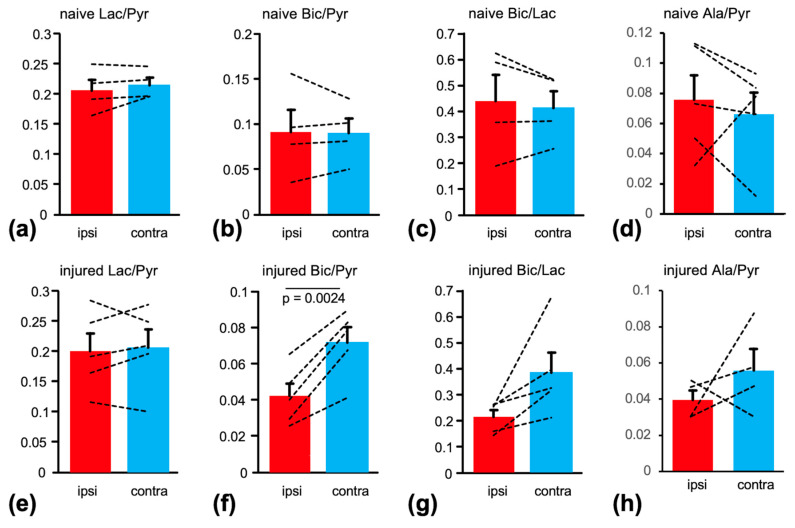
Summary statistics of Lac/Pyr, Bic/Pyr, Bic/Lac, and Ala/Pyr for the comparison between ipsilateral and contralateral ROIs for the naïve (**a**–**d**) and injury (**e**–**h**) groups. Bic/Pyr was lower in the ipsilateral compared to contralateral hemisphere in the injury group whereas no difference was found for any metabolite in naïve animals. (Pyr: pyruvate; Lac: lactate; Bic: bicarbonate; Ala: alanine; ROI: region of interest).

**Figure 5:**
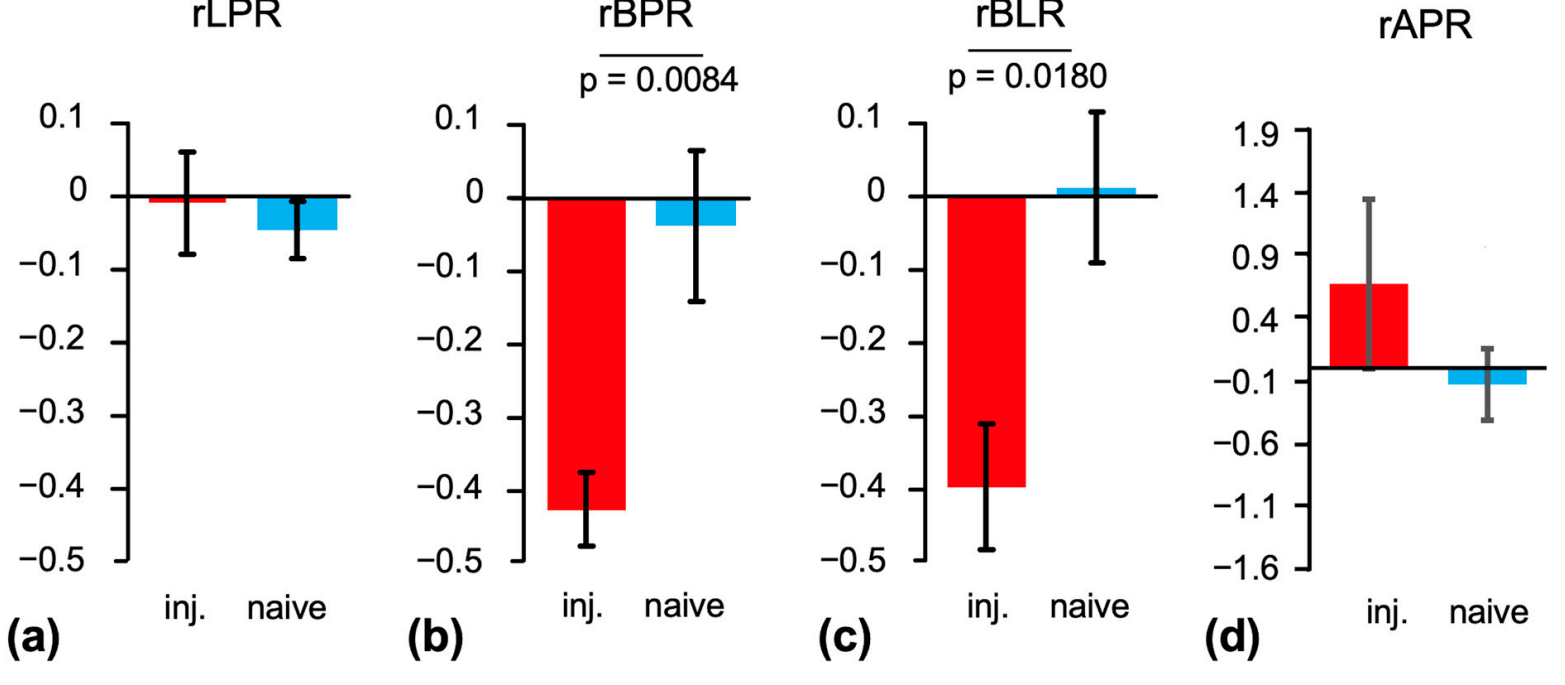
Summary statistics of relative difference between hemispheres for (**a**) Lac/Pyr (rLPR), (**b**) Bic/Pyr (rBPR), (**c**) Bic/Lac (rBLR), and (**d**) Ala/Pyr (rAPR) for the comparison between the two groups. Both rBPR and rBLR are lower in animals after injury compared to naïve animals. (Pyr: pyruvate; Lac: lactate; Bic: bicarbonate; Ala: alanine; rLPR: relative difference of Lac/Pyr; rBPR: relative difference of Bic/Pyr; rBLR: relative difference of Bic/Lac; rAPR: relative difference of Ala/Pyr).

Maps of Lac/Pyr, Bic/Pyr, and Bic/Lac masked to the brain and superimposed onto the T2-weighted MRI from the same animal are shown in Figure 3. Bic/Lac is a metric that reflects aspects of both oxidative and glycolytic energy metabolism, and it has been shown in a rat study of acute TBI to be the metric most sensitive to injury. Furthermore, the ratio of products such as Bic/Lac in preclinical HP ^13^C brain imaging is less sensitive to the level of anesthesia whereas the product-to-substrate ratios can depend on the isoflurane dose as isoflurane is a potent vasodilator [20,21]. The corresponding summary statistics for the two groups are shown in Figure 4. In the injury group, Bic/Pyr was lower at the injury site compared to the contralateral ROI (ipsi: 0.042 ± 0.007 standard error vs. contra: 0.072 ± 0.009; *p* = 0.0024). No difference was found for Lac/Pyr (0.20 ± 0.03 vs. 0.21 ± 0.02), while only a trend was observed for Bic/Lac (0.21 ± 0.03 vs. 0.38 ± 0.08; *p* = 0.0664). There was no difference between hemispheres for any of the metabolite ratios in the naïve animals. When comparing the two groups (Figure 5), the relative difference between hemispheres ([ipsi − contra]/contra) was different for both Bic/Pyr (injury: −0.42 ± 0.5 vs. naïve: −0.03 ± 0.10; *p* = 0.0084) and Bic/Lac (−0.39 ± 0.9 vs. 0.01 ± 0.10; *p* = 0.0180).

**Figure 6:**
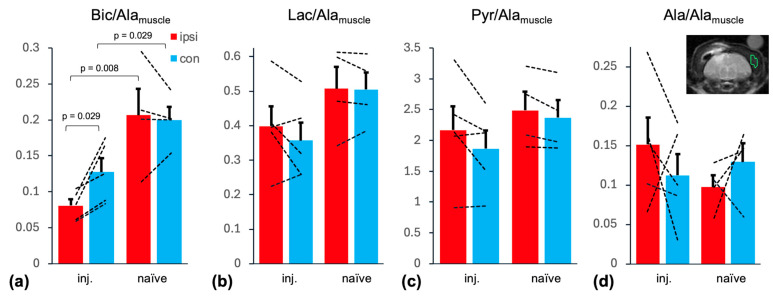
Summary statistics of metabolite levels normalized to muscle Ala for (**a**) Bic, (**b**) Lac, (**c**) Pyr, and (**d**) Ala with comparisons between hemispheres as well as between groups. The location of the muscle ROI is indicated in green in the ^1^H MRI inlet (**d**). In the injury group, Bic/Ala_muscle_ was lower in the ipsilateral compared to contralateral ROI. When comparing the two groups, Bic/Ala_muscle_ in both the ipsilateral and contralateral ROI was lower than in the injury group. (Pyr: pyruvate; Lac: lactate; Bic: bicarbonate; Ala: alanine; ROI: region of interest; MRI: magnetic resonance imaging).

The summary statistics for the metabolites in the brain normalized to Ala from an ROI in the muscle (Ala_muscle_) [22] are shown in Figure 6. When comparing hemispheres in injured animals, the only difference was found for Bic/Ala_muscle_ with a lower level detected in the ipsilateral ROI (ipsi: 0.081 ± 0.009 vs. contra: 0.128 ± 0.019; *p* = 0.029). As was the case when normalizing to brain Pyr in the respective ROI, there was no difference between hemispheres for any of the metabolite ratios in the naïve animals. When comparing the two groups, Bic/Ala_muscle_ was higher in the naïve animals in both the ipsilateral (0.207 ± 0.037; *p* = 0.0078) and contralateral ROI (0.200 ± 0.018; *p* = 0.029).

## 3. Discussion

The presented results demonstrate the feasibility of metabolic imaging of HP [1-^13^C]Pyr in a ferret model of TBI. Eight to ten days post-injury the animals showed lower Bic/Pyr in the ipsilateral hemisphere of the CCI injury compared to the contralateral side. At the same time, there was no difference in Lac/Pyr.

TBI consists of a primary injury involving the direct result of the impact force on the brain. The resulting direct damage to the vasculature, neuronal cell bodies, axons, dendrites, and glia then sets in motion a complex and dynamic chain of events leading to further damage (secondary injury). Early after TBI, ischemia/hypoxia impairs oxidative metabolism and, as a result, the end products of anaerobic metabolism (glycolysis) no longer feed into the TCA cycle, thereby promoting lactate accumulation. Thus, we expect to see an increase in Lac and a decrease in Bic as indicators of acidosis and hypoxia, and indicative of the early stages of the secondary injury. This has been verified in rat models of TBI using metabolic imaging of HP ^13^C pyruvate by us [11,13] and others [14], as well as by a case report on two patients [23]. After restoration of adequate blood flow and cessation of hypoxia, acidosis resolves, and Lac levels normalize. The time course of Lac elevation after TBI seems to be roughly similar across a number of studies: Lac was elevated for at least 7 days in a mouse model [12], but less than 10 days in a rat model [14]. Human case studies are also consistent with the metabolic time course observed in rodent models: Hackett et al. [23] report elevated lactate and lower bicarbonate in one patient who was imaged one day following TBI, while no increase in lactate (despite decreased bicarbonate levels) was observed in another patient who was imaged 6 days after TBI. Here, we performed metabolic imaging 8 to 10 days after TBI and did not observe increased Lac levels, which is consistent with the time courses described above. Our study, as well as prior rodent studies and human case reports have found decreased bicarbonate that persists beyond the increase in lactate. The combined results in mice, rats, and ferrets, as well as human case studies strongly suggest that the metabolic changes following TBI (initial increase in lactate and decrease in bicarbonate production, with a normalization of lactate levels within ~6–10 days) are conserved across species and follow similar time courses. With respect to long-term outcome, potential metabolic changes depend on the severity of injury, ranging from reverting back to normal metabolism to potentially no metabolism in regions of missing/fenestrated tissue [12,13].

Restoration of blood flow to previously hypoxic/ischemic tissue can result in reperfusion injury from excitotoxicity and the formation of reactive oxygen species from the newly resupplied O_2_ (“oxidative stress”). Oxidative stress is linked to mitochondrial dysfunction, leading to the decreased production of bicarbonate, the surrogate marker of oxidative metabolism (reviewed in [24,25,26]). Mitochondrial dysfunction after TBI in rodent models follows a biphasic profile, with severe compromise in the hours immediately following the injury, then a partial recovery followed by a second, sustained phase starting about one day after TBI [27,28]. This particular metabolic derangement persists longer than lactate elevation; in fact, mitochondrial abnormalities and metabolic stress can persist for weeks to months after moderate TBI in mice [29] and rats [28]. Altered Bic levels, as a readout of mitochondrial functioning, may be related to the severity of the primary injury and may serve as a biomarker for later stages of the secondary brain injury after TBI. In this regard, it is intriguing to note that a slightly milder TBI model was used by [14], resulting in a deformation depth of 1 mm and normalized bicarbonate levels after 7 days, while a previous report by us [13] with a deformation depth of 2 mm still found bicarbonate reduction after 7 days. Here, we detected decreased bicarbonate levels at the site of the injury, but not in the contralateral hemisphere 8–10 days post TBI. This was also evidenced in a reduced Bic/Lac ratio, reflecting a relative reduction of aerobic compared to anaerobic metabolism as expected for mitochondrial dysfunction. 

This study demonstrated for the first time the feasibility of metabolic imaging of HP [1-^13^C]Pyr in a ferret model of TBI. The presented results (normal Lac and reduced Bic 8–10 days post TBI) fit well into the framework of oxidative stress as a component of the secondary injury outlasting the increase in lactate production, and are consistent with prior studies using HP ^13^C Pyr imaging in rodents [11,12,13,14].

### Limitations and Future Studies

The experiments described here were intended as part of a proof-of-concept study, in which we successfully demonstrate feasibility and potential utility of [1-^13^C]Pyr metabolic imaging. As a result, treatment groups were quite small (n = 4–5). While we were able to detect some statistically significant effects, the study is underpowered, and we may have missed some true effects. Future research can use this study to estimate the sample size needed for appropriate statistical power (e.g., β = 0.2).

Another important limitation of this study is the lack of a sham control group that underwent surgery but without bCCI procedure. Unfortunately, as the present experiments used a subset of animals from a larger study, additional animals, including shams, were unavailable. However, previous work from our lab suggests that craniotomy surgery does induce a mild brain injury in ferrets [30], making it a poor control for TBI. While naïve animals had not yet undergone surgery, they did receive an additional MRI scan where they were exposed to surgical sedatives and anesthetics at least 1 day prior to metabolic imaging. This exposure was approximately 1 h in duration, which is similar to ferret CCI surgery. As a result, this may be considered an anesthetic exposure control. In addition to isoflurane causing vasodilation, multiple aspects of the specific anesthesia protocol can impact brain metabolism as overserved with HP ^13^C Pyr. Comparing brain metabolism in awake rats to animals under isoflurane, urethane, and medetomidine anesthesia, Hyppönen et al. [31] found that the apparent rate constants for the conversion of Pyr into Lac (k_PL_) and Bic (k_PB_) were highest in awake animals. While there was no difference between awake and isoflurane groups in the ratio of these two metrics, k_PB_/k_PL_ was highest in animals under medetomidine anesthesia compared to all other groups. When comparing isoflurane to pentobarbital, α-chloralose, and morphine anesthesia, Marjańska et al. [32] report the highest k_PB_ for morphine whereas k_PL_ was highest in the isoflurane group. Furthermore, Healicon et al. [33] found that the composition of the carrier gas impacts the results in rats under isoflurane anesthesia, with Bic/Pyr and Bic/Lac highest when using a 60/40% mix of O_2_/N_2_O compared to a 90/10% mixture or pure O_2_. Nevertheless, the sham group would have allowed assessment of the effects of craniotomy surgery on the detected metabolite levels in the brain. The fact that Ala was detected in the brain ROIs suggests contributions from scalp tissue to the ROIs in the brain, considering that at least in rat brains the activity of alanine transaminase is approximately 50 times lower than LDH [34]. However, at the same time, no difference in Ala/Pyr between hemispheres (ipsi: 0.076 ± 0.016 vs. contra: 0.066 ± 0.016; *p* = 0.55) or in the relative difference between groups (injury: 0.67 ± 0.67 vs. naïve: −0.13 ± 0.28; *p* = 0.35) was detected. There was also no difference in Ala/Ala_muscle_ between hemispheres or groups. Additionally, Bic/Ala_muscle_ was lower even in the contralateral ROI in injured compared to naïve animals. Although it is possible that the blast injury affected pyruvate metabolism in the muscle tissue close to the contralateral ROI, taken together, these findings suggest that differences found in Bic levels were most likely due to differences in brain metabolism rather than only due to lower scalp signal. However, future studies should be conducted at higher resolution in order to reduce partial volume effects. Similarly, all animals underwent under-vehicle blast, which is a whole-body injury. As a result, both hemispheres were impacted and ipsilateral vs. contralateral comparisons should be interpreted cautiously. Nevertheless, assuming that the blast injury affects brain metabolism qualitatively the same way as the CCI injury, a comparison based on relative differences between hemispheres underestimates the metabolic differences between the injured and naïve animals. This injury type is military-relevant; blast injury accounts for approximately 60% of military TBI [35]. However, the translational value of this model to civilian populations may be limited, as impact injury is far more common.

The metabolic data were acquired as a single-time point measurement where quantification of metabolite ratios is more sensitive to user-induced experimental variations, e.g., duration of the Pyr injection, compared to a time-resolved acquisition that also permits quantifying the metabolite kinetics using kinetic models [36,37]. However, metrics based on comparisons between hemispheres in the same animal are less sensitive to variations in timing. Although ferrets are small enough to be imaged in small animal MRI scanners, the experiments were performed on a clinical scanner as it was equipped with sufficiently large ^1^H/^13^C radio frequency (RF) coil. Due to lower gradient performance the phase-encoded free induction decay chemical shift imaging (FIDCSI) sequence was chosen to achieve the resolution and field-of-view (FOV) necessary for ferrets. A scanner with a high-performance gradient system would not only allow a dynamic acquisition but also higher spatial resolution, which reduces partial volume effects and potential contributions from scalp tissue. Additionally, higher gradient performance would facilitate extension to 3D imaging [21], which would be especially beneficial in longitudinal studies as it allows registration of the imaging data across time points. As such, goals of future studies include following the time course of dysregulated energy metabolism from the acute stage over a longer period and correlating the severity and duration with behavioral outcomes. Additionally, investigating the perturbations of brain energy metabolism in a model that more closely reproduces the major forms of clinical TBI such as falls, vehicular accidents, and sports-related collisions would increase the translational relevance. The closed head injury model of engineered rotational acceleration (CHIMERA) model of TBI has been used in ferrets and could be well-suited for this type of study [38].

In conclusion, this study demonstrates the feasibility of using metabolic imaging of HP [1-^13^C]Pyr to measure perturbations in brain energy metabolism in a novel ferret model of TBI. Developing quantitative biomarkers for dysregulated metabolism after TBI in this highly translatable animal model could contribute to our understanding of injury mechanisms and aid in identifying new therapeutic targets. When translated into the clinic, these imaging biomarkers could lead to improved diagnosis and provide valuable information for patient management.

## 4. Materials and Methods

Seven male ferrets (~10 weeks old; Marshall Bioresources, North Rose, NY, USA) were used for this study. Animals were pair-housed until injury and then individually housed to avoid wound dehiscence from rough-and-tumble play until suture removal two weeks later, at which time they were reunited with their cage mate. Animal care staff provided standardized environmental enrichment including ferret-specific treats, toys, bedding, and burrows. Prior to metabolic imaging, uninjured (naïve) animals underwent baseline behavior testing and structural/functional MRI. After injury and the completion of the post-injury metabolic imaging experiments, ferrets underwent additional behavior testing, structural/functional MRI, and histopathologic analysis of brain injury as part of a larger study—these results will be reported elsewhere. 

### 4.1. Blast and Controlled Cortical Impact

Ferrets were sedated 15–30 min before the blast with an intraperitoneal injection of 0.05 mg/kg dexmedetomidine (Dexdomitor, Zoetis, Parsippany, NJ, USA) and anesthetized with 4% isoflurane in a 45% Air/O_2_ mixture for 4–5 min in an induction chamber. Prone ferrets were then secured into a custom polycarbonate cylinder restraint (12.7 × 40.6 cm^2^) affixed upon two 2.5 cm thick aluminum blast plates (40.6 × 38 cm^2^) which were separated by a 6 mm rubber pad. Pentaerythritol tetranitrate (PETN; 2.5 g) was submerged 51 mm in a polyurea coated steel tank (1.2 × 0.6 × 1.2 m^3^) with a 5 mm air gap between the water surface and bottom plates to avoid shockwave transfer directly into the plate [39,40]. A licensed explosive expert detonated the explosive, causing the plates to travel ~1 m vertically, guided by poles at the corner of the plates. Immediately after the blast, ferrets were removed from the restrainer and proceeded to controlled cortical impact surgery. 

Animals were re-anesthetized with 4% isoflurane as above and then were maintained on 1.5–2% isoflurane via nosecone. Ferrets were secured in a stereotactic frame with ear bars (Kopf, Tujunga, CA, USA) atop a homeothermic blanket system (Harvard Apparatus, Holliston, MA, USA), which maintained rectal temperature at 37 ± 0.5 °C. Heart rate, respiratory rate, and blood oxygen saturation (SpO2) were monitored (Biopac Systems, Inc., Goleta, CA, USA) for the duration of the procedure (~1 h). Animals were aseptically prepared, and a midline dorsal incision (3–4 cm over the calvarium) was made to visually identify the supraorbital crest junction. The center of the 6–7 mm craniotomy was positioned 15 mm caudal and 6 mm lateral to the junction, between the coronal, lambdoid, and sagittal sutures. The impact was made using a CCI device (Impact One, Leica Biosystems, Wetzlar, Germany) with a 5 mm beveled piston at 6.0 mm depth with a 6.0 m/s velocity, and 50 ms dwell time. Surgifoam (Ethicon, Raritan, NJ, USA) was placed over the craniotomy and secured with dental acrylic; once the acrylic was dry, the incision was sutured. Atipamezole (0.05 mg/kg) was provided intraperitoneally to reverse dexmedetomidine sedation, if needed. Buprenorphine (0.01–0.05 mg/kg, s.c.) was administered as analgesia every 8–12 h for 3 days with the first dose administered just prior to surgery. Animals were then monitored twice daily for the first three days, then daily for the duration of the experiment. 

### 4.2. Animal Handling for MRI

Prior to imaging, ferrets were sedated with dexmedetomidine and anesthesia was induced with isoflurane as above. They were maintained on 1% isoflurane anesthesia via nose cone and placed in sternal recumbency. A microwavable heating pad was applied to either the lateral saphenous or cephalic vein for approximately 3 min to allow for vessel dilation and greater visibility. The site was then clipped and cleaned with 70% ethanol and a heparinized 22–24 gauge, 1 in catheter/needle combination was inserted and guided into the vein. The catheter was secured and flushed with heparinized saline. Ferret skin is tough, and their veins are difficult to see/palpate. Given that these animals were part of a larger experiment that may have been impacted by other injuries, efforts to catheterize were abandoned if the technician was unsuccessful after two attempts in each vein.

The animal was placed inside the magnet, and an MR-compatible small-animal monitoring and gating system was used to monitor respiration rate and body temperature (SA Instruments, Stony Brook, NY, USA). Body temperature was maintained at 37 ± 0.5 °C using a circulating water blanket (Stryker T). The anesthesia was kept at 1.5% isoflurane in 100% O_2_ at 1 L/min throughout the whole imaging session.

### 4.3. Pyruvate Polarization and Injection

The solution of HP Pyr was generated as previously described [11]. Briefly, ~100 mg [1-^13^C]pyruvic acid and 15 mM trityl radical were hyperpolarized for a minimum of 3 h in a 5T GE SPINLab (Research Circle Technology, Niskayuna, NY, USA) followed by rapid dissolution with water and neutralization with sodium hydroxide. The final solution had a concentration of ~114 mM [1-^13^C]Pyr with pH ~7.3. Within approximately 30 to 40 s after dissolution, a bolus of ~9.5 mL/kg was injected by hand into the catheterized vein over ~25 s followed by 0.5 mL saline to clear the injection line.

### 4.4. MR Acquisitions

Due to catheterization challenges, of the seven animals, two animals were imaged before injury only, three animals were imaged after injury only, and two animals were imaged before and after injury. The experiments were performed on a 3T GE Discovery 750w scanner (GE Healthcare, Waukesha, WI, USA) using a dual-tuned ^1^H/^13^C quadrature RF coil (80 mm diameter, USA Instruments Inc., Aurora, OH, USA). Single-shot fast spin-echo (FSE) ^1^H MR images with an FOV of 120 × 60 mm^2^ (512 × 192 matrix), 3 mm slice thickness with an echo time (TE) and repetition time (TR) of 76.8 ms and 1500 ms, respectively, were acquired in all three principal axes as anatomical reference for prescribing the ^13^C magnetic resonance spectroscopic imaging (MRSI) experiments. Additionally, dual-echo FSE images (FOV = 12 cm, 256 × 192 matrix, 1 mm slice thickness, TE1/TE2/TR = 41.8/83.5/3000 ms, echo train length = 8) were acquired matching the location of the ^13^C MRSI prescription. Thirty seconds after start of the injection of HP pyruvate (as described above), ^13^C MRSI data were acquired from a single axial (animal coronal) slice covering the location of the injury using a 2D phase-encoded FIDCSI sequence (8 mm slice, 60 mm^2^ FOV, 256 points at 5 kHz spectral bandwidth, 16 × 16 matrix, concentric k-space encoding with circularly reduced k-space sampling, variable flip angle scheme, 16 s acquisition time) [41].

### 4.5. Data Processing and Statistical Analysis

Reconstruction and post-processing of the ^13^C MRSI were performed with custom-written software using MATLAB (Version R2020a, Mathworks, Natick, MA, USA). The spatial k-space data were apodized with a Hanning window and zero-filled by a factor of 2. In the spectroscopic dimension, backward linear prediction was used to fill in the missing data points due to the delay between excitation and start of data acquisition. Afterwards, the data were apodized with a Voigt function with linewidths of 25 Hz and −5 Hz for the Gaussian and Lorentzian components, respectively, and zero-filled by a factor 8. Three-dimensional fast Fourier transform resulted in a 32 × 32 map of spectra. Metabolic maps for Pyr, Lac, Bic, and Ala were calculated by integrating the signal within a 44 Hz interval around each peak in absorption mode. Maps for metabolite ratios were also calculated. ROIs were placed in the ipsilateral (injured) and contralateral (non-injured) hemispheres (cf. Figure 2) and the metabolite signals in the spectra averaged over each ROI were quantified by fitting a Gaussian curve to the respective peak.

Statistical analyses were performed using SigmaPlot (Version 15, Grafiti, Palo Alto, CA, USA). One-way analysis of variance (ANOVA) was used to assess differences between naïve and injured groups. Assumptions of normality and equal variance were assessed with the Shapiro–Wilks and Brown–Forsythe methods prior to data analysis. The Kruskal–Wallis one-way ANOVA on Ranks with Dunn’s post-hoc test was used in the event of failed normality tests. Paired t-tests were also used to compare differences between the ipsilateral and contralateral sides. Statistical significance was defined as *p* < 0.05. Given the limited number of pre-planned comparisons in this exploratory study, corrections for multiple comparisons were not applied [42].

## Data Availability

The original contributions presented in this study are included in the article. Further inquiries can be directed to the corresponding author.
